# Vascular Health Assessment of The Hypertensive Patients (VASOTENS) Registry: Study Protocol of an International, Web-Based Telemonitoring Registry for Ambulatory Blood Pressure and Arterial Stiffness

**DOI:** 10.2196/resprot.5619

**Published:** 2016-06-29

**Authors:** Stefano Omboni, Igor N Posokhov, Gianfranco Parati, Alberto Avolio, Anatoly N Rogoza, Yulia V Kotovskaya, Giuseppe Mulè, Maria Lorenza Muiesan, Iana A Orlova, Elena A Grigoricheva, Ernesto Cardona Muñoz, Parounak H Zelveian, Telmo Pereira, João Manuel Peixoto Maldonado

**Affiliations:** ^1^ Italian Institute of Telemedicine Clinical Research Unit Solbiate Arno Italy; ^2^ Hemodynamic Laboratory Ltd Nizhniy Novgorod Russian Federation; ^3^ Department of Cardiology and Department of Medicine and Surgery Istituto Auxologico Italiano and University of Milano-Bicocca Milano Italy; ^4^ Faculty of Medicine and Health Sciences Department of Biomedical Sciences Macquarie University Sydney Australia; ^5^ Russian Cardiology Research and Production Complex Department of New Diagnostic Methods Moscow Russian Federation; ^6^ Peoples' Friendship University of Russia Department of Internal Medicine Propaedeutics Moscow Russian Federation; ^7^ Centro di Riferimento Regionale per l'Ipertensione Arteriosa, Policlinico Paolo Giaccone Unità Operativa di Nefrologia ed Ipertensione Palermo Italy; ^8^ Spedali Civili and University of Brescia Dipartimento di Scienze Mediche e Chirurgiche and Medicina 2 Brescia Italy; ^9^ Lomonosov Moscow State University Clinic Moscow Russian Federation; ^10^ South Ural State Medical University Department of Outpatient Therapy and Clinical Pharmacology Chelyabinsk Russian Federation; ^11^ University of Guadalajara Department of Physiology Guadalajara Mexico; ^12^ L.A. Ohanesyan Institute of Cardiology Center of Preventive Cardiology Yerevan Armenia; ^13^ Escola Superior de Tecnologia da Saúde de Coimbra , Instituto Politécnico de Coimbra Coimbra Portugal; ^14^ Clínica da Aveleira Instituto de Investigação e Formação Cardiovascular Coimbra Portugal

**Keywords:** blood pressure telemonitoring, arterial stiffness, pulse wave velocity, augmentation index, central aortic pressure, hypertension

## Abstract

**Background:**

Hypertension guidelines recommend ambulatory blood pressure (ABP), central aortic pressure (CAP), and pulse wave velocity (PWV) as parameters for estimating blood pressure (BP) control and vascular impairment. Recent advances in technology have enabled devices to combine non-invasive estimation of these parameters over the 24-hour ABP monitoring. However, currently there is limited evidence on the usefulness of such an approach for routine hypertension management.

**Objective:**

We recently launched an investigator-initiated, international, multicenter, observational, prospective study, the Vascular health Assessment Of The Hypertensive patients (VASOTENS) Registry, aimed at (1) evaluating non-invasive 24-hour ABP and arterial stiffness estimates (through 24-hour pulse wave analysis, PWA) in hypertensive subjects undergoing ambulatory blood pressure monitoring (ABPM) for clinical reasons; (2) assessing the changes in estimates following treatment; (3) weighing the impact of 24-hour PWA on target organ damage and cardiovascular prognosis; (4) assessing the relationship between arterial stiffness, BP absolute mean level and variability, and prognosis; and (5) validating the use of a 24-hour PWA electronic health (e-health) solution for hypertension screening.

**Methods:**

Approximately 2000 subjects, referred to 20 hypertension clinics for routine diagnostic evaluation and follow-up of hypertension of any severity or stage, will be recruited. Data collection will include ABPM, performed with a device allowing simultaneous non-invasive assessment of 24-hour CAP and arterial stiffness (BPLab), and clinical data (including cardiovascular outcomes). As recommended by current guidelines, each patient will be followed-up with visits occurring at regular intervals (ideally every 6 months, and not less than once a year depending on disease severity). A Web-based telemedicine platform (THOLOMEUS) will be used for data collection. The use of the telemedicine system will allow standardized and centralized data collection, data validation by experts and counseling to remote centers, setup and maintenance of the Registry, and prompt data analysis.

**Results:**

First follow-up results are expected to be available in the next 2 years.

**Conclusions:**

The results of the VASOTENS Registry will help define the normalcy thresholds for current and future indices derived from 24-hour PWA, according to outcome data, and will also provide supporting evidence for the inclusion of this type of evaluation in hypertension management.

**Trial registration:**

Clinicaltrials.gov NCT02577835; https://clinicaltrials.gov/ct2/show/NCT02577835 (Archived by WebCite at http://www.Webcitation.org/6hzZBKY2Q)

## Introduction

### Literature Review

Central aortic pressure (CAP) and pulse wave velocity (PWV) are independent predictors for the development of cardiovascular (CV) diseases [1-3]. Due to the large amount of epidemiological evidence for its predictive value for CV events, carotid-femoral PWV is currently highly indicated and useful for stratification of total CV risk, and considered the “gold standard” measurement of arterial stiffness [1,4,5]. Pulse-wave analysis (PWA), including evaluation of CAP, and Augmentation Index (AIx), may also provide additional information concerning wave reflections and may be useful for risk stratification and evaluation of the effectiveness of treatment, but more evidence is needed before recommending the routine clinical use of these vascular indices [1,5].

The most widely adopted methods for evaluating pulse waveforms are those based on applanation tonometry and transfer functions [1,6,7]. Notwithstanding the consistent evidence for superior and independent prognostic value, with respect to conventional office BP of either indices of central hemodynamics and stiffness assessed in controlled office condition at rest [2,3,8,9], currently there are no studies that evaluated the long-term predictive ability for CV events of vascular indices (ie, PWV, AIx, and CAP), measured in dynamic conditions over the 24-hours by ambulatory blood pressure monitoring (ABPM). However, some incomplete evidence is available from cross-sectional studies. For example, in 629 patients with diabetes, 24-hour aortic systolic blood pressure (SBP) was higher than in the 86 control patients, and increased with diabetic complications, being more strongly associated to complications than peripheral 24-hour SBP [10]. In the SAFAR Study [11,12], both 24-hour aortic and brachial SBP were superior to conventional office BP measurements in predicting BP-related cardiac damage (left ventricular hypertrophy and left ventricular diastolic dysfunction) in 230 subjects (75% having arterial hypertension). In the same study, 24-hour ambulatory central SBP was also more closely associated with left ventricular hypertrophy than 24-hour ambulatory brachial SBP. With respect to arterial stiffness, Aissopou et al [13] found that ambulatory aortic PWV, estimated by an operator-independent method, provided additional information to carotid-femoral PWV regarding the association of arterial stiffness with the retinal vessel calibers. Elsurer and Afsar [14] found that in 339 hypertensive patients with chronic kidney disease, serum uric acid was significantly correlated with both 24-hour PWV and AIx. However, serum uric acid was independently associated with AIx only. Maloberti and colleagues [15] studied 19 children with Williams-Beuren syndrome, a genetic disorder involving the elastin gene and adversely affecting arterial function and found that sick children showed higher heart rate and AIx values at night than age-matched controls, suggesting an abnormal sympathetic cardiovascular control and an increase in small arteries resistance. The limited evidence from the literature is completed by two longitudinal studies. The Ambulatory Central Aortic Pressure (AmCAP) study described a significant CAP lowering effect on both day-time and night-time with a 12-week treatment based on either aliskiren (300 mg) or telmisartan (80 mg) administered once-daily [16]. Interestingly, this study also showed relatively higher values of nocturnal aortic than brachial BP. Karpetas et al [17] and Koutroumbas et al [18] showed a gradual interdialytic increase in ambulatory CAP and AIx, and to less extent in PWV, in 153 patients with end stage renal disease treated with dialysis.

There is previous evidence that non-invasive assessment of 24-hour arterial stiffness and central hemodynamics in daily life conditions measured by the device used in this study (BPLab device, BPLab GmbH, Schwalbach am Taunus, Hessen, Germany) may help in assessing the arterial function impairment in hypertensive patients. We recently showed larger 24-hour CAP and peripheral AIx values in 661 patients with hypertension compared to 142 normotensive controls [19]. In a subgroup of 137 patients with hypertension, a significantly positive correlation was found between 24-hour PWV and left ventricular mass index (LVMI) [20]. In another study, Kuznetsova et al [21] provided age- and gender-specific reference diagnostic values for 24-hour PWV, AIx, and CAP in 467 normotensive volunteers. Minyukhina and colleagues [22] observed a reduction in 24-hour PWV one week after kidney transplantation in 41 patients with end-stage renal disease, with a return to pre-transplant values after 20 weeks. Finally, in another study the combination of hypertension and chronic obstructive pulmonary disease was associated with an increased ambulatory peripheral and central aortic pressure, while this was not the case for isolated essential hypertensive subjects and normotensive controls [23].

### Rationale

Recent advances in technology enabled devices to combine non-invasive estimation of CAP and arterial stiffness in ambulatory conditions over the 24-hours, based on the oscillometric method [24-28]. Such techniques are affordable and may allow a comfortable, accurate, repeated, and prolonged estimation of arterial stiffness and central hemodynamics over the 24-hours in daily life conditions by ABPM. Recent studies seem to indicate reliability and feasibility of ambulatory arterial stiffness and hemodynamics evaluation based on analysis of brachial oscillograms [8,29,30]. However, at present, there is limited evidence on the clinical usefulness of such an approach and much has to be done to prove its actual benefit in the daily clinical management of hypertension. In particular, there are very few data on the long-term prognostic and clinical value of 24-hour ambulatory CAP and arterial stiffness estimation, since most studies performed so far were not sufficiently long lasting or had a cross-sectional design [11-23]. At the moment, some associations with CV complications have been demonstrated for 24-hour CAP, but not for PWV and AIx. In addition, since different algorithms are used by the different ambulatory devices, non-invasive estimation of central hemodynamics and arterial stiffness appears to be device- and/or technique-dependent, and thus results obtained with one or the other device cannot be easily confronted and interpreted.

To provide further insight on the matter, we created a large database (or registry) of ABPM recordings obtained with the BPLab monitor, which is able to determine CAP, PWV, and AIx, over the 24-hours, based on a clinically validated technology of PWA of oscillometric BP measurements [26-28]. The choice of this device was made not only because of its proved accuracy and clinical reliability, but also because of its compatibility with the Web-based telemedicine platform THOLOMEUS (Biotechmed Ltd., Somma Lombardo, Varese Italy) [31], which enables easy data collection and communication within such a large worldwide network of study centers.

This paper summarizes the study protocol (final version, dated 20/02/2015, available as an online supplement to this paper) (Multimedia Appendix 1) that has been prepared following the recommendations contained in the SPIRIT statement [32], the most appropriate checklist for the publication of protocol papers of observational, non-randomized, prospective studies in the initial stage (Multimedia Appendix 2).

### Study Objectives

The VASOTENS (Vascular health Assessment Of The Hypertensive patients) Registry aims at evaluating the clinical value and the prognostic impact of 24-hour ambulatory non-invasive estimation of arterial stiffness and central hemodynamics by PWA in patients with hypertension undergoing an ABPM for clinical reasons in hypertension clinics. Specific study objectives include (1) the evaluation of 24-hour PWV, AIx and CAP in hypertensive patients over consecutive ABPMs performed at regular intervals, as recommended by current guidelines, for a minimum of 2 years (main study objective); (2) the evaluation of the changes in BP and arterial stiffness estimates following treatment initiation or modification, according to current guidelines; (3) the assessment of the impact of non-invasive arterial stiffness estimation on cardiac, vascular and renal damage and patient’s CV prognosis (fatal and non-fatal events); (4) the definition of the normal thresholds for PWV, AIx and CAP, in hypertensive patients, according to outcome data; and (5) the definition of the relationship between arterial stiffness, BP absolute level and BP variability, and outcomes.

The outcome-based results provided by the VASOTENS Registry will help establish a worldwide network of certified centers performing ambulatory PWA and will help validate and foster the use of the 24-hour PWA electronic health (e-health) solution for hypertension screening and follow-up. Ultimately, the study-based evidence of the clinical relevance of 24-hour non-invasive central arterial stiffness and hemodynamics assessment may help favor the inclusion of such evaluations among the standard procedures made available in hypertension centers, as well as practical recommendations for improving hypertension management and control.

### Trial Design

The VASOTENS Registry is an international, multicenter, observational, non-randomized, prospective study.

## Methods

### Study Setting

A minimum of 20 hypertension centers will be involved worldwide, each providing at least 100 participants, in order to allow recruitment of a sufficiently consistent sample size able to demonstrate the study objectives. A list of participating centers grouped by countries is shown in Multimedia Appendix 3. Initially, hospitals from Italy and Russia will be enrolled because of their proximity to the study coordinators. Attempts will be made to select investigators among active members of international and national hypertension societies, including membership in the bodies specifically dedicated to blood pressure and arterial stiffness measurement. This will ensure high quality standards of data collection and facilitate the dissemination of information on the project and its findings.

### Eligibility Criteria

Participants referred to the study centers for routine diagnostic evaluation of hypertension or established hypertensive patients will be eligible for inclusion in the study. Individuals fulfilling eligibility criteria (Textbox 1), whose data are contained in existing databases collected by the participating centers, and who are regularly followed-up at the center will have priority for enrolment.

**Study eligibility criteria.**

Criteria

- Inclusion criteria
  - Male and female
  - Age ≥18 years
  - Participants referred to routine diagnostic evaluation for hypertension of any severity or stage, or established hypertensive patients
  - Good quality ABPM performed for clinical reasons with a BPLab device
    - Availability of individual measurements for ABPM on a bpw file (BPLab format)
    - Data directly uploaded on the telemedicine platform of the study
  - Availability of basic clinical information (see Textbox 2)
  - Availability of a signed informed consent form
- Exclusion criteria

- Age <18 years
- Atrial fibrillation, frequent ectopic beats, second or third degree atrioventricular blocks, or other conditions which might make difficult or unreliable the automatic BP measurement with the oscillometric technique
- Upper arm circumference <22 cm
- Pregnancy

However, naïve hypertensive participants will also be enrolled, provided that an ABPM is required for evaluating their potential hypertension status, according to current recommendations [5,33]. Once enrolled, participants will be visited every 6 or 12 months at the study centers (dependant on disease severity) for a minimum follow-up of 2 years, and submitted to the procedures detailed in the next sections.

### Study Procedures

The project will not involve any type of diagnostic evaluation or pharmacological intervention and the investigators will be free to manage the patients included in the Registry according to the requirements of clinical practice and current guidelines [5]. However, as guidelines recommend, each patient will be followed-up with visits occurring at regular intervals: ideally every 6 months, and not less than once a year, for a minimum follow-up of 2 years. The investigators will also be free to use the information yielded by the ABPM tests for the clinical management of their patients. At each study visit, an ABPM by the BPLab device will be performed and patient’s clinical data, such as family history, anthropometric data, habits, past and current diseases, therapies, office BP, and laboratory tests, including evaluation of target organ damage, will be collected and entered on the electronic case report form (e-CRF) located on the study website. A detailed list of the clinical data to be collected during the study is itemized in Textbox 2.

**Basic demographic and clinical information to be collected during the study.**

Demographic and clinical information

- Age
- Gender
- Height (cm)
- Weight (kg)
- Ethnicity
- Superficial distance between jugulum and symphysis (surrogate of aortic length; cm)
- Waist circumference (cm)
- Smoking status
- Alcohol drinking
- Coffee or tea drinking
- Dyslipidemia (yes/no and indication on treatment)
- Diabetes (yes/no and indication on treatment)
- Diagnosis of hypertension (yes/no and indication on treatment)
- Family history of premature CV disease
- Medical history with particular regard to previous and/or concurrent CV diseases
- Office BP (mmHg) and heart rate (bpm) obtained in the same treatment condition as ABPM
- Electrocardiogram (ECG) indication on left ventricular hypertrophy, Sokolow–Lyon and Cornell index)
- Left ventricular mass index (LVMI, as g/m^2^) at echocardiogram
- When available, diameter of the aorta (aortic annulus, root and sinotubular junction, in cm) and/or cardiac output (as L/min), assessed by the echocardiogram
- Intima-media thickness (IMT, mm) at carotid ultrasonography
- When available, ankle-brachial index
- Microalbuminuria (as mg/24h) or albumin-creatinine ratio (mg/g), and serum creatinine (g/dL), with subsequent calculation of estimated glomerular filtration rate (eGFR) by the Cockroft-Gault equation
- When available, PWV (m/s), Aix (%), and CAP (mmHg) taken during the office visit with a validated device different from the BPLab device (eg, Sphygmocor or Complior)

#### Ambulatory Blood Pressure Monitoring

Twenty-four hour ABPM will be performed with the BPLab device, which has been found to be accurate for estimation of both BP and vascular indices in properly conducted validation studies [26,27,34-36]. A description of the technique used to non-invasively assess central hemodynamics and arterial stiffness by the BPLab device is detailed in a separate section.

Current guidelines will be followed for proper recording performance [33,37,38]. In order to reduce patient’s discomfort and to ensure a reliable minimum number of BP measurements for the subsequent data analysis (particularly for the evaluation of BP variability), the device will be programmed to measure BP at least every 20 minutes during the day (providing a minimum of 3 readings per hour) and at least 30 minutes during the night (providing a minimum of 2 readings per hour). Whenever possible, recordings will start between 8 am and 11 am, in order to standardize data collection and comparisons. The monitoring cuff will be placed on the non-dominant arm, the lower edge 2 cm above elbow bend, with the bladder centered on the upper arm, to ensure uniform compression and decompression during inflation and deflation. In order to allow the proper evaluation of PWV, the length of the aorta will be derived by measuring the distance from the sternal notch (jugulum) to the upper edge of the pubic bone (symphysis). In the case of obese patients, this superficial morphological distance will be adjusted by using the frontal projection in standing position. Up to 2 BP test readings will be triggered manually before the device is activated for automatic measurements in order to test its proper functioning. Two sequential conventional (office) BP and heart rate readings will also be taken in the sitting position at the time of ABPM placement (with the same BPLab device or with a validated automatic or manual BP measuring device), and recorded on the e-CRF. Patients will be instructed to keep the arm still and to avoid any movement during each automatic BP measurement. Patients will be free to attend to their usual daily activities during ABPM (avoiding strenuous exercise). They will have to complete a diary in which daily activities (ie, time of sleeping, time of meals) will be reported together with the time of occurrence of unusual events or poor night sleep quality. The patient will come back to the outpatient clinic on the second day of the recording (after at least 24 hours) to remove the monitor. Shortly after device removal, the recording will be downloaded to a computer using the telemedicine Web platform of the project. The investigator will obtain the results from the Web-based analysis software and verify each recording for compliance with quality criteria (see below for details). In case of a bad quality recording the investigator will have to repeat the recording as soon as possible, preferably within 2 days.

#### Pulse-Wave Analysis

The oscillometric BPLab device will also allow measurements of ambulatory arterial stiffness and central hemodynamics by recording pulsatile pressure changes at the brachial artery level. Briefly, during BP measurement, the pressure waveforms in the cuff are recorded during a step-by-step deflation and then digitalized and stored in the device memory. When data are uploaded on the Web-based telemedicine platform, the software processes the signal using proprietary mathematical algorithms. These are based on a specially developed hemodynamic model to get the PWV and transfer function that utilizes a modification in a certain frequency range within the acquired pulse signal to derive the aortic pressure wave, and thus to assess CAP and AIx. A detailed description of the methodology may be found elsewhere [26,28,30]. As aforementioned, the accuracy of the BPLab device for the assessment of vascular indices has been validated in studies against a non-invasive gold standard [26,27].

#### Web-Based Telemedicine Platform

**Figure 1:**
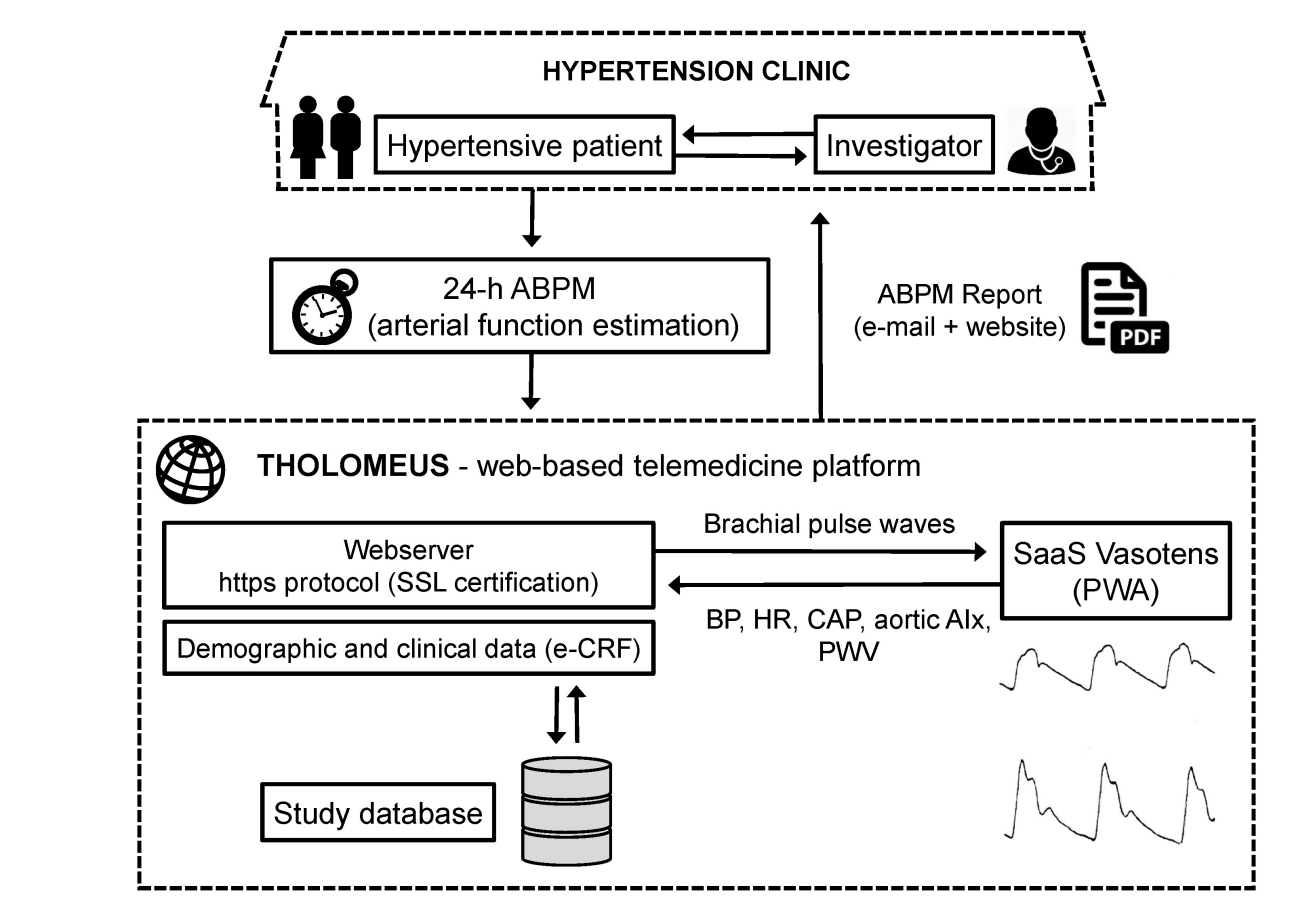
Workflow of the THOLOMEUS Web-based telemedicine system used in the VASOTENS Registry.

Data contained in existing electronic databases or data of newly enrolled subjects fulfilling the inclusion criteria will be uploaded and entered on the study website. These data will include ABPM measures (peripheral or brachial BP, CAP, and arterial stiffness) obtained with a BPLab device and clinical data. Data collection will be ensured by a certified Web-based telemedicine platform (THOLOMEUS, Biotechmed Ltd., Somma Lombardo, Varese Italy) available on the Tholomeus website [31]. The choice of using an e-health tool for study management is based on the potential of such a solution to allow standardized and centralized data collection, prompt data validation and analysis, effective study monitoring and auditing, easy and real-time distribution of software updates, and bug corrections. It will also help provide advanced screening options for the patients with hypertension through a worldwide network of expert centers connected together. ABPM data will be uploaded on the website as bpw files (original file format of the standard analysis software of BPLab device, for centers already using such software) or by plugging the ABPM device to the computer through a *Universal Serial Bus* (USB) cable. ABPM data will be transmitted to the website and analyzed in real-time with production of an electronic report (as an Acrobat Reader pdf file) sent by email to the investigator and simultaneously published on the user-restricted area of the website. The Web-based telemedicine platform is complemented by an e-CRF, which will allow entering main patient’s clinical data into the study database. Access to the website and e-CRF will be granted through authentication with username and password according to local data protection and privacy regulations. A schematic diagram of the workflow of the Web-based telemedicine platform is shown in Figure 1.

### Ethics

The study will be conducted according to Good Clinical Practice guidelines and the Declaration of Helsinki [39]. The data collection will start in each center only after approval or notification (depending on local laws) of the study protocol and amendments (if any) by the independent ethics committees of the centers. All participants meeting inclusion criteria and not meeting exclusion criteria will be fully informed about the study design and purposes, and asked to give a written informed consent if willing to participate. All patient-related information is subject to medical confidentiality and to the local data protection acts. Data will be pseudonymized before any aggregate analysis. This means that main data useful to identify the patient will be replaced with a unique number and thus the patient’s identity will not be disclosed to third parties, except the promoter.

### Data Quality Control

Given its observational nature, no formal monitoring of the study is foreseen for this study. However, electronic data verification will be done remotely by a data manager who will get in touch with the investigators and, when needed, will ask the investigator to correct the erroneous data or complete missing data on the e-CRF. The investigator will be required to verify and check that the information provided on the e-CRF is as precise and accurate as possible. The procedures for data monitoring and verification will be ensured by the presence of logical checks and range (defined a priori) for the different variables and by automatic identification of inconsistencies by the e-CRF used to manage the database. The controls and related corrections will be made on the e-CRF directly by the investigator on the website. Since no standardized or centralized analysis of laboratory tests will be done, except for ABPM, particular attention will be dedicated to check the congruency of data collected through ultrasonography, ECG, and biochemistry.

### Dissemination Activities

An important part of the study-related activities will be aimed at disseminating the knowledge on the correct use of ambulatory CAP and arterial stiffness estimation in clinical practice and in research, and thus at achieving a possibly standardized and widespread use of this integrated technology. Manuscripts reporting main study results and documents embedding specific recommendations on the use of the technique will be developed. All publications related to the study results will be prepared by the study coordinators with the support of the scientific committee and will include any investigator significantly contributing to the success of the study. A complete list of investigators will be provided at the end of each manuscript. The original study protocol (with appendices) can be downloaded from, and any future study results will be published on the VASOTENS study website.

### Statistical Methods

#### Primary Outcome Measures

The primary study endpoint will consist of the calculations of the average 24-hour values of PWV, CAP, and AIx during the study. The main time points will be the baseline versus the end of the study, corresponding to 2 years following enrolment, but averages will be computed for each study visit occurring during the follow-up.

#### Secondary Outcome Measures

The endpoints considered secondary study variables and evaluated according to the same timeline applied to the primary outcome measures are shown in Textbox 3.

**Secondary outcome measures.**

Outcome measures

- Average 24-hour brachial (or peripheral) SBP and DBP
- 24-hour brachial SBP and DBP variability estimated by:
  - Unweighted standard deviation: the standard deviation of 24-hour mean value of brachial SBP and DBP [40]
  - Weighted standard deviation: the standard deviation of the average of all brachial SBP and DBP values during day-time and night-time, with weights corresponding to the duration of day-time and night-time [41]
  - Average real variability (ARV): the mean of the successive absolute differences between adjacent brachial SBP and DBP values over the 24-hours [42]
- Cardiac damage, defined by the presence of cardiac hypertrophy, as determined by echocardiography (LVMI >115 g/m^2^ in men and >95 g/m^2^ in women according to the recommendations of the American Society of Echocardiography) [43,44] or ECG (Sokolow-Lyon index >3.5 mV + R in aVL >1.1 mV or Cornell voltage duration product >244 mV*ms) [5]
- Vascular damage, defined by the presence of carotid wall thickening or plaque (intima media thickness, IMT >0.9 mm) at ultrasonography and, if available, by an ankle-brachial index (ABI) <0.9 [5]
- Renal damage, defined by microalbuminuria (30-300 mg/24 h) or albumin–creatinine ratio (30–300 mg/g) (preferentially on morning spot urine) or reduced estimated glomerular filtration rate (eGFR <60 ml/min/1.73 m^2^) [5]
- Cardiovascular fatal or non-fatal events: death or hospitalization for congestive heart failure, myocardial infarction, angina, stroke or cerebrovascular accident, renal failure, or other cardiovascular diseases

#### Sample Size

Given the observational nature of the study and the lack of precedent studies with a similar design and objectives, it is difficult to define a proper sample size. According to the number of subjects enrolled and followed-up in previous cross-sectional or prospective trials [10,11,14-21], and considering the number of participants usually needed to collect a sufficient number of clinical outcomes in longitudinal population studies based on ABPM [45], a minimum number of 2000 participants has been considered for the present study. Ideally, such a sample size will be able to provide consistent outcome-based information on the clinical relevance of 24-hour PWA.

#### Statistical Analysis

Analysis will be performed on all participants with valid ABPM recordings at study entry and during the follow-up. Principal derived ABPM variables and arterial stiffness measures will be immediately calculated once the data will be uploaded on the website and their quality verified. Analysis of 24-hour recordings will be preceded by removal of artifacts according to previously described editing criteria [37,38]. Valid recordings will be considered those with (1) an interval between measurements not exceeding 30 minutes during the whole 24-hours; (2) a recording duration of at least 24-hours; (3) at least 70% of expected number of readings; and (4) at least 20 valid readings during the day-time and 7 during the nigh-time. Average brachial and central SBP and DBP, and arterial stiffness indices (PWV and AIx) will be computed by averaging all the individual readings over the 24-hours, and separately for the day-time and night-time subperiods, and for each hour of the recording. Measures of BP variabilities (unweighted standard deviation, weighted standard deviation and ARV) will also be computed based on individual readings. Other ABPM variables of interest (eg, nocturnal BP fall, morning surge, etc) will be subsequently defined in the framework of sub-analyses based on the Registry data and calculated according to procedures specifically defined.

Basic descriptive statistics will be provided for all variables with calculations of absolute and relative frequencies for categorical variables and calculation of average value, standard deviation, and minimum and maximum for continuous variables. The relationship between BP and arterial stiffness estimates, and organ damage and prognosis will be evaluated by appropriate parametric or non parametric tests, depending on the type of data distribution (normal or non-normal). The occurrence of any cardiovascular event during the study will be evaluated by the Kaplan-Meier method. Time-to-event curves will be drawn and the survival analysis will be performed according to the Cox proportional hazard model to analyze predictors of outcomes. Data management and analysis will be carried out by SPSS for Windows version 20. A *P*<0.05 will be considered as the minimum level of statistical significance.

## Results

Enrolment of patients in the first study centers started in October 2015. The first data analysis is expected to be performed by the end of 2017 or early 2018.

## Discussion

The VASOTENS Registry is an international, multicenter, observational, non-randomized, prospective study devised to evaluate the clinical impact and usefulness of 24-hour PWA for hypertension management.

### Expected Contributions

With respect to all of the studies briefly reviewed in this paper, our Registry may offer, for the first time, the possibility to shed light on the role of 24-hour ambulatory central hemodynamics and stiffness as predictors of cardiovascular outcomes. The study results may help determine whether the clinical value of ABPM might be further increased by incorporating information on ambulatory CAP and stiffness. The results of the VASOTENS Registry will help define the normalcy thresholds for current and future indices derived from 24-hour PWA, according to outcome data. They will also provide supporting evidence for the inclusion of such evaluations in recommendations on hypertension management and its possible impact on the general population health state. Thanks to this study, an important lack of knowledge will be worked-out and the foundation for future studies with a more robust design could be hopefully laid.

### Limitations

The non-randomized uncontrolled nature of the study and the rather wide selection criteria may increase the risk of obtaining heterogeneous and poorly powered results. In addition, the fact that patients will be recruited in hypertension centers may result in a potential selection bias: the sample being unrepresentative or not fully representative of the general population of patients with hypertension. Despite these important limitations, we think that since the study is carried out in a real-life setting and that it is a longitudinal long-term outcome-driven study, it represents an important added value.

### Dissemination Strategy

An important component of this study is disseminating the knowledge on correct use of ambulatory CAP and arterial stiffness estimation and to help create an e-health network for a standardized and widespread use of this hypertension screening tool. In order to achieve this, apart from data collection, several disseminating activities are required. An exchange of knowledge between participating centers will be achieved by the cooperation of investigators in preparing a possibly unified methodology of ABPM data collection and analysis and by jointly addressing methodological issues that may arise during the project. Data collected in the Registry will foster the performance of studies aimed at optimizing a possible clinical application of non-invasive ambulatory arterial stiffness estimation. The study will help provide instructions on appropriate ambulatory arterial stiffness monitoring methodology to other physicians, particularly to intermediate level centers, not necessarily experts in ABPM use and arterial stiffness determination. A major task of the consortium will be to provide these participants with accurate information on correct methodology and interpretation of such data, in order to support them in case of difficulties and to monitor the correctness of the use of the methodology in these centers during the project. The study findings will favor preparation of specific recommendations on the use and clinical application of ABPM integrated with arterial stiffness evaluation. The study will also ensure cooperation between international and national scientific societies in the area related to ABPM and arterial stiffness monitoring. This will facilitate the dissemination of information on the project and its findings and will also allow an interaction with writing committees involved in the preparation of guidelines pertinent to this area

### Conclusions

The results of the data collected at baseline and during regular follow-up of hypertensive patients in the VASOTENS Registry will help define the normalcy thresholds for current and future indices derived from 24-hour PWA, according to outcome data. They will also provide supporting evidence on the clinical usefulness of such a technological approach, based on telemedicine, for the screening and follow-up of the vascular function status of the patients with hypertension.

## Appendix

Multimedia Appendix 1 VASOTENS Registry Study Protocol.

Multimedia Appendix 2 Updated SPIRIT checklist.

Multimedia Appendix 3 List of centers.

## Abbreviations

ABP: ambulatory blood pressure
ABPM: ambulatory blood pressure monitoring
Alx: Augmentation Index
BP: blood pressure
CAP: central aortic pressure
CV: cardiovascular
ECG: electrocardiogram
e-CRF: electronic case report form
LVMI: left ventricular motility injury
PWA: pulse-wave analysis
PWV: pulse wave velocity
SBP: systolic blood pressure
VASOTENS: Vascular health ASsessment Of The HypertENSive Patients

## References

[1] Laurent S, Cockcroft J, Van Bortel L, Boutouyrie P, Giannattasio C, Hayoz D, Pannier B, Vlachopoulos C, Wilkinson I, Struijker-Boudier H, European Network for Non-invasive Investigation of Large Arteries (2006). Expert consensus document on arterial stiffness: methodological issues and clinical applications. Eur Heart J.

[2] McEniery CM, Cockcroft JR, Roman MJ, Franklin SS, Wilkinson IB (2014). Central blood pressure: current evidence and clinical importance. Eur Heart J.

[3] Vlachopoulos C, Aznaouridis K, Stefanadis C (2010). Prediction of cardiovascular events and all-cause mortality with arterial stiffness: a systematic review and meta-analysis. J Am Coll Cardiol.

[4] Ben-Shlomo Y, Spears M, Boustred C, May M, Anderson SG, Benjamin EJ, Boutouyrie P, Cameron J, Chen C, Cruickshank JK, Hwang S, Lakatta EG, Laurent S, Maldonado J, Mitchell GF, Najjar SS, Newman AB, Ohishi M, Pannier B, Pereira T, Vasan RS, Shokawa T, Sutton-Tyrell K, Verbeke F, Wang K, Webb DJ, Willum HT, Zoungas S, McEniery CM, Cockcroft JR, Wilkinson IB (2014). Aortic pulse wave velocity improves cardiovascular event prediction: an individual participant meta-analysis of prospective observational data from 17,635 subjects. J Am Coll Cardiol.

[5] Mancia G, Fagard R, Narkiewicz K, Redón J, Zanchetti A, Böhm M, Christiaens T, Cifkova R, De BG, Dominiczak A, Galderisi M, Grobbee DE, Jaarsma T, Kirchhof P, Kjeldsen SE, Laurent S, Manolis AJ, Nilsson PM, Ruilope LM, Schmieder RE, Sirnes PA, Sleight P, Viigimaa M, Waeber B, Zannad F, Task FM (2013). 2013 ESH/ESC Guidelines for the management of arterial hypertension: the Task Force for the management of arterial hypertension of the European Society of Hypertension (ESH) and of the European Society of Cardiology (ESC). J Hypertens.

[6] Kim DH, Braam B (2013). Assessment of arterial stiffness using applanation tonometry. Can J Physiol Pharmacol.

[7] Nelson MR, Stepanek J, Cevette M, Covalciuc M, Hurst RT, Tajik AJ (2010). Noninvasive measurement of central vascular pressures with arterial tonometry: clinical revival of the pulse pressure waveform?. Mayo Clin Proc.

[8] Vlachopoulos C, Aznaouridis K, O'Rourke MF, Safar ME, Baou K, Stefanadis C (2010). Prediction of cardiovascular events and all-cause mortality with central haemodynamics: a systematic review and meta-analysis. Eur Heart J.

[9] Zhang Y, Agnoletti D, Safar ME, Wang J, Topouchian J, Xu Y, Protogerou AD, Blacher J (2013). Comparison study of central blood pressure and wave reflection obtained from tonometry-based devices. Am J Hypertens.

[10] Theilade S, Lajer M, Hansen TW, Joergensen C, Persson F, Andrésdottir G, Reinhard H, Nielsen SE, Lacy P, Williams B, Rossing P (2013). 24-hour central aortic systolic pressure and 24-hour central pulse pressure are related to diabetic complications in type 1 diabetes - a cross-sectional study. Cardiovasc Diabetol.

[11] Protogerou AD, Argyris AA, Papaioannou TG, Kollias GE, Konstantonis GD, Nasothimiou E, Achimastos A, Blacher J, Safar ME, Sfikakis PP (2014). Left-ventricular hypertrophy is associated better with 24-h aortic pressure than 24-h brachial pressure in hypertensive patients: the SAFAR study. J Hypertens.

[12] Zhang Y, Kollias G, Argyris AA, Papaioannou TG, Tountas C, Konstantonis GD, Achimastos A, Blacher J, Safar ME, Sfikakis PP, Protogerou AD (2015). Association of left ventricular diastolic dysfunction with 24-h aortic ambulatory blood pressure: the SAFAR study. J Hum Hypertens.

[13] Aissopou EK, Argyris AA, Nasothimiou EG, Konstantonis GD, Tampakis K, Tentolouris N, Papathanassiou M, Theodossiadis PG, Papaioannou TG, Stehouwer CD, Sfikakis PP, Protogerou AD (2016). Ambulatory aortic stiffness is associated with narrow retinal arteriolar caliber in hypertensives: the SAFAR study. Am J Hypertens.

[14] Elsurer R, Afsar B (2014). Serum uric acid and arterial stiffness in hypertensive chronic kidney disease patients: sex-specific variations. Blood Press Monit.

[15] Maloberti A, Cesana F, Hametner B, Dozio D, Villa P, Hulpke-Wette M, Schwarz A, Selicorni A, Wassertheurer S, Mancia G, Giannattasio C (2015). Increased nocturnal heart rate and wave reflection are early markers of cardiovascular disease in Williams-Beuren syndrome children. J Hypertens.

[16] Williams B, Lacy PS, Baschiera F, Brunel P, Düsing R (2013). Novel description of the 24-hour circadian rhythms of brachial versus central aortic blood pressure and the impact of blood pressure treatment in a randomized controlled clinical trial: the Ambulatory Central Aortic Pressure (AmCAP) study. Hypertension.

[17] Karpetas A, Sarafidis PA, Georgianos PI, Protogerou A, Vakianis P, Koutroumpas G, Raptis V, Stamatiadis DN, Syrganis C, Liakopoulos V, Efstratiadis G, Lasaridis AN (2015). Ambulatory recording of wave reflections and arterial stiffness during intra- and interdialytic periods in patients treated with dialysis. Clin J Am Soc Nephrol.

[18] Koutroumbas G, Georgianos PI, Sarafidis PA, Protogerou A, Karpetas A, Vakianis P, Raptis V, Liakopoulos V, Panagoutsos S, Syrganis C, Passadakis P (2015). Ambulatory aortic blood pressure, wave reflections and pulse wave velocity are elevated during the third in comparison to the second interdialytic day of the long interval in chronic haemodialysis patients. Nephrol Dial Transplant.

[19] Omboni S, Posokhov IN, Rogoza AN (2015). Evaluation of 24-hour arterial stiffness indices and central hemodynamics in healthy normotensive subjects versus treated or untreated hypertensive patients: a feasibility study. Int J Hypertens.

[20] Posokhov IN, Kulikova NN, Starchenkova IV, Grigoricheva EA, Evdokimov VV, Orlov AV, Rogoza AN, BPLab-Vasotens RC (2014). The “Pulse Time Index of Norm” highly correlates with the left ventricular mass index in patients with arterial hypertension. Vasc Health Risk Manag.

[21] Kuznetsova TY, Korneva VA, Bryantseva EN, Barkan VS, Orlov AV, Posokhov IN, Rogoza AN, BPLab-Vasotens RC (2014). The 24-hour pulse wave velocity, aortic augmentation index, and central blood pressure in normotensive volunteers. Vasc Health Risk Manag.

[22] Minyukhina IE, Lipatov KS, Posokhov IN (2013). Analysis of 24-hour pulse wave velocity in patients with renal transplantation. Int J Nephrol Renovasc Dis.

[23] Aksenova TA, Gorbunov VV, Parkhomenko IV (2013). [24-hour monitoring central aortic pressure in patients with hypertensive disease and concomitant chronic obstructive pulmonary disease]. Klin Med (Mosk).

[24] Wassertheurer S, Kropf J, Weber T, van der Giet M, Baulmann J, Ammer M, Hametner B, Mayer CC, Eber B, Magometschnigg D (2010). A new oscillometric method for pulse wave analysis: comparison with a common tonometric method. J Hum Hypertens.

[25] Luzardo L, Lujambio I, Sottolano M, da Rosa A, Thijs L, Noboa O, Staessen JA, Boggia J (2012). 24-h ambulatory recording of aortic pulse wave velocity and central systolic augmentation: a feasibility study. Hypertens Res.

[26] Rogoza AN, Kuznetsov AA (2012). Central aortic blood pressure and augmentation index: comparison between Vasotens and SphygmoCor technology. RRCC.

[27] Kotovskaya YV, Kobalava ZD, Orlov AV (2014). Validation of the integration of technology that measures additional “vascular” indices into an ambulatory blood pressure monitoring system. Med Devices (Auckl).

[28] Posokhov IN (2013). Pulse wave velocity 24-hour monitoring with one-site measurements by oscillometry. Med Devices (Auckl).

[29] Protogerou AD, Argyris A, Nasothimiou E, Vrachatis D, Papaioannou TG, Tzamouranis D, Blacher J, Safar ME, Sfikakis P, Stergiou GS (2012). Feasibility and reproducibility of noninvasive 24-h ambulatory aortic blood pressure monitoring with a brachial cuff-based oscillometric device. Am J Hypertens.

[30] Ageenkova OA, Purygina MA (2011). Central aortic blood pressure, augmentation index, and reflected wave transit time: reproducibility and repeatability of data obtained by oscillometry. Vasc Health Risk Manag.

[31] THOLOMEUS (TelemedicineHOme teLemOnitoring for MEdical sUrveillance of chronic diSeases).

[32] Chan A, Tetzlaff JM, Altman DG, Laupacis A, Gøtzsche PC, Krleža-Jerić K, Hróbjartsson A, Mann H, Dickersin K, Berlin JA, Doré CJ, Parulekar WR, Summerskill WS, Groves T, Schulz KF, Sox HC, Rockhold FW, Rennie D, Moher D (2013). SPIRIT 2013 statement: defining standard protocol items for clinical trials. Ann Intern Med.

[33] O'Brien E, Parati G, Stergiou G, Asmar R, Beilin L, Bilo G, Clement D, de la Sierra A, de LP, Dolan E, Fagard R, Graves J, Head GA, Imai Y, Kario K, Lurbe E, Mallion J, Mancia G, Mengden T, Myers M, Ogedegbe G, Ohkubo T, Omboni S, Palatini P, Redon J, Ruilope LM, Shennan A, Staessen JA, vanMontfrans G, Verdecchia P, Waeber B, Wang J, Zanchetti A, Zhang Y, European Society of Hypertension Working Group on Blood Pressure Monitoring (2013). European Society of Hypertension position paper on ambulatory blood pressure monitoring. J Hypertens.

[34] Koudryavtcev SA, Lazarev VM (2011). Validation of the BPLab(®) 24-hour blood pressure monitoring system according to the European standard BS EN 1060-4:2004 and British Hypertension Society protocol. Med Devices (Auckl).

[35] Dorogova IV, Panina ES (2015). Comparison of the BPLab® sphygmomanometer for ambulatory blood pressure monitoring with mercury sphygmomanometry in pregnant women: validation study according to the British Hypertension Society protocol. Vasc Health Risk Manag.

[36] Ledyaev MY, Stepanova OV, Ledyaeva AM (2015). Validation of the BPLab(®) 24-hour blood pressure monitoring system in a pediatric population according to the 1993 British Hypertension Society protocol. Med Devices (Auckl).

[37] Parati G, Omboni S, Palatini P, Rizzoni D, Bilo G, Valentini M, Rosei EA, Mancia G (2008). Italian society of hypertension guidelines for conventional and automated blood pressure measurement in the office, at home and over 24 hours. High Blood Press Cardiovasc Prev.

[38] Omboni S, Palatini P, Parati G, Working Group on Blood Pressure Monitoring of the Italian Society of Hypertension (2015). Standards for ambulatory blood pressure monitoring clinical reporting in daily practice: recommendations from the Italian Society of Hypertension. Blood Press Monit.

[39] World MA (2013). World Medical Association Declaration of Helsinki: ethical principles for medical research involving human subjects. JAMA.

[40] Mancia G, Ferrari A, Gregorini L, Parati G, Pomidossi G, Bertinieri G, Grassi G, di RM, Pedotti A, Zanchetti A (1983). Blood pressure and heart rate variabilities in normotensive and hypertensive human beings. Circ Res.

[41] Bilo G, Giglio A, Styczkiewicz K, Caldara G, Maronati A, Kawecka-Jaszcz K, Mancia G, Parati G (2007). A new method for assessing 24-h blood pressure variability after excluding the contribution of nocturnal blood pressure fall. J Hypertens.

[42] Mena L, Pintos S, Queipo NV, Aizpúrua JA, Maestre G, Sulbarán T (2005). A reliable index for the prognostic significance of blood pressure variability. J Hypertens.

[43] Devereux RB, Reichek N (1977). Echocardiographic determination of left ventricular mass in man. Anatomic validation of the method. Circulation.

[44] Lang RM, Bierig M, Devereux RB, Flachskampf FA, Foster E, Pellikka PA, Picard MH, Roman MJ, Seward J, Shanewise JS, Solomon SD, Spencer KT, Sutton MS, Stewart WJ, Chamber Quantification Writing Group, American Society of Echocardiography's GuidelinesStandards Committee, European Association of Echocardiography (2005). Recommendations for chamber quantification: a report from the American Society of Echocardiography's Guidelines and Standards Committee and the Chamber Quantification Writing Group, developed in conjunction with the European Association of Echocardiography, a branch of the European Society of Cardiology. J Am Soc Echocardiogr.

[45] Gu Y, Thijs L, Li Y, Asayama K, Boggia J, Hansen TW, Liu Y, Ohkubo T, Björklund-Bodegård K, Jeppesen J, Dolan E, Torp-Pedersen C, Kuznetsova T, Stolarz-Skrzypek K, Tikhonoff V, Malyutina S, Casiglia E, Nikitin Y, Lind L, Sandoya E, Kawecka-Jaszcz K, Imai Y, Mena LJ, Wang J, O'Brien E, Verhamme P, Filipovsky J, Maestre GE, Staessen JA, International Database on Ambulatory blood pressure in relation to Cardiovascular Outcomes (IDACO) Investigators (2014). Outcome-driven thresholds for ambulatory pulse pressure in 9938 participants recruited from 11 populations. Hypertension.

