# Supplementary material for: Vascular Health Assessment of The Hypertensive Patients (VASOTENS) Registry: Study Protocol of an International, Web-Based Telemonitoring Registry for Ambulatory Blood Pressure and Arterial Stiffness
Source: JMIR Res Protoc. 2016 Jun 29;5(2):e137. doi: 10.2196/resprot.5619 (PMC4945820; doi:10.2196/resprot.5619)
Supplement: Multimedia Appendix 1 [file resprot_v5i2e137_app1.pdf]

1.0 TITLE PAGE

INTERNATIONAL REGISTRY FOR AMBULATORY BLOOD PRESSURE AND ARTERIAL STIFFNESS TELEMONITORING

VASOTENS (**V**ascular health **AS**sessment **O**f **T**he hypert**ENS**ive patients) **Registry**

Final Version - 20/02/2015

Project Coordinator: Dr. Stefano Omboni
Clinical Research Unit
Italian Institute of Telemedicine
Via Colombera 29
21048 Solbiate Arno (Varese)
Italy

Co-coordinator: Dr. Igor Posokhov
Hemodynamic Laboratory
PO Box 69
603009 Nizhniy Novgorod
Russia

CONFIDENTIALITY STATEMENT

The information in this document is privileged or confidential and may not be disclosed unless such disclosure is required by law. In any event, persons to whom the information is disclosed must be informed that the information is privileged or confidential and may not be further disclosed by them. These restrictions on disclosure will apply equally to all future information supplied to you which is indicated as privileged or confidential.

2.0 TABLE OF CONTENTS

1.0 TITLE PAGE **1**
2.0 TABLE OF CONTENTS **2**
3.0 RESPONSIBILITIES **3**
4.0 SYNOPSIS **4**
5.0 LIST OF ABBREVIATIONS **7**
6.0 INTRODUCTION **8**
7.0 PROJECT OBJECTIVES **8**
8.0 OVERALL PROJECT DESIGN AND PLAN **8**
9.0 SELECTION OF PROJECT POPULATION **8**
9.1 INCLUSION CRITERIA 9
9.2 EXCLUSION CRITERIA 9
10.0 PROJECT ACTIVITIES **9**
10.1 CREATION OF RESEARCH NETWORK AND DATA COLLECTION 9
10.2 ABPM DATA ANALYSIS 10
10.3 DISSEMINATION ACTIVITIES 10
11.0 STATISTICAL METHODS **10**
12.0 INVESTIGATORS AND PROJECT ADMINISTRATIVE STRUCTURE **11**
12.1 PROJECT COORDINATORS 11
12.2 SCIENTIFIC COMMITTEE AND OTHER PROJECT STRUCTURES 11
12.2.1 Steering Committee *11*
12.2.2 Scientific Committee *11*
12.2.3 National Coordinators *12*
12.2.4 Scientific and Organizing Secretariat *12*
12.3 INVESTIGATORS 12
12.4 CRO 12
13.0 ETHICS **12**
13.1 INDEPENDENT ETHICS COMMITTEE OR INSTITUTIONAL REVIEW BOARD 12
13.2 SUBJECT INFORMATION AND CONSENT 12
14.0 DATA MANAGEMENT **13**
14.1 ELECTRONIC CASE REPORT FORM (E-CRF) 13
14.2 PROJECT MONITORING 14
14.3 DATA QUALITY CONTROL AND ASSURANCE 14
15.0 INSURANCE **14**
16.0 DISCONTINUATION OF THE PROJECT **14**
17.0 STATEMENT OF CONFIDENTIALITY **15**
18.0 REPORTING **15**
19.0 DATA ACCESS AND PUBLICATION POLICY **15**
20.0 PROTOCOL AMENDMENTS **15**
21.0 REFERENCE LIST **16**
22.0 APPENDICES **18**
APPENDIX 1 – AMBULATORY BLOOD PRESSURE MONITORING PROCEDURES 19
APPENDIX 2 – PATIENT INFORMATION SHEET AND INFORMED CONSENT FORM 20
APPENDIX 3 – INVESTIGATOR'S APPROVAL PAGE 23

3.0 RESPONSIBILITIES

Project Coordinator

Stefano Omboni
Clinical Research Unit
Italian Institute of Telemedicine
Via Colombera 29
21048 Solbiate Arno (Varese)
Italy

Co-coordinator:

Igor Posokhov
Hemodynamic Laboratory
PO Box 69
603009 Nizhniy Novgorod
Russia

Steering Committee

Stefano Omboni (Italy), Gianfranco Parati (Italy), Igor Posokhov (Russia), Anatoli Rogoza (Russia)

Scientific Committee

Stefano Omboni (Italy), Gianfranco Parati (Italy), Igor Posokhov (Russia), Anatoli Rogoza (Russia), Yulia Kotovskaya (Russia)

Study centers:

Italian centers (initially 10-15)
Russian centers (initially 10-15)

4.0 SYNOPSIS

**Project title**	**INTERNATIONAL REGISTRY FOR AMBULATORY BLOOD PRESSURE AND ARTERIAL STIFFNESS TELEMONITORING**
**Project name**	**VASOTENS** (**V**ascular health **AS**sessment **O**f **T**he hypert**ENS**ive patients) **REGISTRY**
**Scientific Coordinator**	Dr. Stefano Omboni (Italy)<br>Clinical Research Unit<br>Italian Institute of Telemedicine<br>Via Colombera 29<br>21048 Solbiate Arno (Varese)<br>Italy
**Co-coordinator**	Dr. Igor Posokhov (Russia)<br>PO Box 69<br>603009 Hemodynamic Laboratory<br>Nizhniy Novgorod<br>Russia
**Project centers**	At least 30 Hypertension centers will be involved in the project, each one providing at least 100 patients, which will be followed-up over time. Centers will be selected in different countries, initially in Europe, starting from Italy and Russia. Mandatory criteria of a center to be included in the study are the availability in the facility of a BPLab ABPM monitor, the potential for providing and properly following-up the number of patients required by the protocol, the availability of Internet connection, regular access to the web and human resources to upload ABPM and clinical data.
**Date of starting of the project**	Launch of the project (first investigators' meeting): June 2015<br>First patient uploaded into the database: September 2015
**Objectives**	The project has several practical objectives and questions to be answered. These will be:<br>• The evaluation of non-invasive ambulatory blood pressure and arterial stiffness estimates (through pulse wave analysis, PWA) in hypertensive subjects undergoing an ambulatory blood pressure monitoring (ABPM) for clinical reasons in the selected centers<br>• The evaluation of the changes in blood pressure and arterial stiffness estimates following treatment initiation according to current guidelines<br>• The assessment of the impact of non-invasive arterial stiffness estimation on target organ damage and patient's cardiovascular (CV) prognosis<br>• The definition of the normalcy thresholds for pulse wave velocity (PWV), augmentation index (AI), and other current and future indices derived from PWA in hypertensive subjects, according to outcome data<br>• The definition of the relationship between arterial stiffness, blood pressure absolute level and blood pressure variability, and outcomes<br>• The setup of a worldwide network of centers performing ambulatory PWA, and the validation and promotion of the use of such technique for hypertension screening and follow-up<br>• The provision of evidence of the clinical relevance of non-invasive arterial stiffness assessment, in order to favor the inclusion of such evaluation in recommendations on hypertension management
**Methodology**	International, multicenter, observational, prospective project
**Population**	The registry will include data from subjects fulfilling the inclusion criteria and whose data are contained in existing databases collected by the participating centers and who are regularly followed-up at the center. New subjects can be enrolled for this project, but they must be submitted to ABPM because it is required for evaluating their hypertension status, according to current recommendations
**Inclusion criteria**	• Male and female subjects<br>• Age $\geq$18 years<br>• Subjects referred to routine diagnostic evaluation for hypertension or established hypertensive subjects<br>• ABPM performed for clinical reasons with a BPLab device<br>• The minimum validity requirements for inclusion of the ABPM in the study are:<br>− Interval between measurements not exceeding 30 minutes

	– At least 70% of expected number of readings<br>– At least 20 valid readings during the day-time and 7 during the nigh-time<br>• Availability of individual measurements for ABPM on a .bpw file (BPLab format) or data directly downloaded on the telemedicine platform of the study (see below for details)<br>• Availability of basic demographic and clinical information:<br>– Age<br>– Gender<br>– Height<br>– Weight<br>– Ethnicity<br>– Superficial distance between jugulum and symphysis (surrogate of aortic length)<br>– Waist circumference<br>– Smoking status<br>– Alcohol drinking<br>– Coffee or tea drinking<br>– Dyslipidemia (yes/no and indication on treatment)<br>– Diabetes (yes/no and indication on treatment)<br>– Diagnosis of hypertension (yes/no and indication on treatment)<br>– Family history of premature CV disease<br>– Medical history, with particular regard to previous and/or concurrent CV diseases<br>– Office blood pressure and heart rate obtained in the same treatment condition as ABPM<br>– Left ventricular mass index (LVMI) at echocardiogram<br>– When available, diameter of the aorta (aortic annulus, root and sinotubular junction) and/or cardiac output, assessed by the echocardiogram<br>– Intima-media thickness (IMT) at carotid ultrasonography<br>– ECG (indication on left ventricular hypertrophy, Sokolow–Lyon and Cornell index)<br>– When available, ankle-brachial index<br>– Microalbuminuria and serum creatinine (calculation of estimated glomerular filtration rate - eGFR)<br>– When available, pulse wave velocity (PWV), augmentation index (AI) and central blood pressure taken during the office visit with a validated device different for BPLab Vasotens (e.g. Sphygmocor or Complior)<br>• Availability of a signed informed consent form
**Exclusion criteria**	• Age <18 years<br>• Atrial fibrillation, frequent ectopic beats, second or third degree atrioventricular blocks, or other conditions which might make difficult or unreliable the automatic blood pressure measurement with the oscillometric technique<br>• Upper arm circumference <22 cm<br>• Pregnancy
**Data collection**	Data contained in existing electronic databases or data of newly enrolled subjects fulfilling the inclusion criteria will be uploaded/entered on the study website. These data will include ABPM measures (blood pressure and arterial stiffness) obtained with a BPLab device and clinical data. ABPM data will be provided as .bpw files or directly uploaded by linking the ABPM device to the website through a personal computer.<br>Data collection will be ensured by a dedicated web-based telemedicine platform, including an electronic case report form (e-CRF). The e-CRF will allow collection of patient's clinical data, such as family history, anthropometric data, habits, past and current diseases, therapies, office BP, and laboratory tests, including evaluation of target organ damage.<br>The project does not involve any type of intervention related to the study and the physicians will manage the patients included in the Registry according to the requirements of clinical practice and current guidelines. However, as recommended by current guidelines, each patient will be followed-up with visits occurring at regular intervals (ideally every 6 months, and not less than once a year). The

	physicians will also be free to use the ambulatory blood pressure data in the clinical management of the patients.<br>The telemedicine system will allow a) standardized and centralized data collection, b) data validation by experts and counselling to remote centers, c) setup and maintenance of the Registry.
**Statistical methods**	Standard variables (systolic and diastolic blood pressure, pulse pressure and heart rate), central systolic and diastolic blood pressure, and arterial stiffness indices (PWV and AI) will be averaged for the whole 24-hour period, and separately for the day-time and night-time subperiods. Additionally, hourly averages will be computed, and in case of blood pressure, variability will be evaluated by calculation of weighted standard deviation and average real variability.<br>Other variables of interest will be defined in the course of the study and calculated subsequently according to the procedures defined in a specific statistical analysis plan.<br>The occurrence of any CV event (death or hospitalization for congestive heart failure, myocardial infarction, angina, stroke or cerebrovascular accident, renal failure, etc.) during the study will be evaluated by the Kaplan-Meier method. Time-to-event curves will be drawn and the survival analysis will be performed according to the Cox proportional hazard model, which will allow to analyze predictors of outcomes.<br>Relationship between blood pressure and arterial stiffness estimates and organ damage and prognosis will be evaluated.

5.0 LIST OF ABBREVIATIONS

ABPM	=	Ambulatory Blood Pressure Monitoring
AI	=	Augmentation Index
CRO	=	Contract Research Organization
CV	=	Cardiovascular
ECG	=	Electrocardiogram
e-CRF	=	electronic CRF
eGFR	=	Estimated Glomerular Filtration Rate
IMT	=	Intima Media Tickness
LVMI	=	Left Ventricular Mass Index
PWA	=	Pulse Wave Analysis
PWV	=	Pulse Wave Velocity

6.0 INTRODUCTION

In recent years, great emphasis has been placed on the role of arterial stiffness and central blood pressure as independent predictors for the development of cardiovascular (CV) diseases [1-3]. Consequently, the assessment of arterial stiffness and central hemodynamics is recommended as additional tests for the clinical evaluation of hypertensive patients (based on history, physical examination and findings from routine laboratory tests), particularly for those at risk for CV complications [4].
Regional and local arterial stiffness may be measured directly, and non-invasively, at various sites along the arterial tree, by assessing Pulse Wave Velocity (PWV) and Augmentation Index (AI) [1]. Central BPs are derived from non-invasive techniques of measurement of radial or carotid pulses [5].
The most widely employed methods for evaluating pulse waveforms are those based on applanation tonometry and transfer functions, although recently oscillometric ambulatory blood pressure monitoring (ABPM) devices using specific algorithms for pulse wave analyses (PWA) have been proposed for assessing arterial stiffness [6-9]. At present, oscillometry is an affordable technique, and may allow a comfortable, accurate, repeated and prolonged estimation of arterial stiffness and central hemodynamics over the 24-hours in daily life conditions [9]. The most recent studies seem to indicate reliability and feasibility of ambulatory arterial stiffness evaluation based on analysis of brachial oscillograms [10-12].
However, very few information is available on the prognostic value of ambulatory central blood pressure [13,14], and no information at all is provided by any study on the clinical value of 24-hour arterial stiffness estimation (PWV and AI). For this reason, we decided to create a large database (registry) of ABPM recordings obtained with a non-invasive device, able to determine central BP and various indices of arterial stiffness (mainly PWV and AI) over the 24-hours, based on a clinically validated technology of PWA of oscillometric BP measurements, integrated in the ambulatory BP monitor [10,12,13]. Specifically, this project aims at creating an international network of centers performing ABPM and arterial stiffness monitoring, in order to evaluate the impact of such estimates on the clinical outcome of hypertensive patients. The results of the data collected at baseline and during regular follow-up of hypertensive patients will help to provide evidence on the clinical usefulness of such technologies for the screening and follow-up of the hypertensive patients.

7.0 PROJECT OBJECTIVES

The specific project objectives are:

- The evaluation of non-invasive ambulatory blood pressure and arterial stiffness estimates (through PWA) in hypertensive subjects undergoing an ABPM for clinical reasons in the selected centers
- The evaluation of the changes in blood pressure and arterial stiffness estimates following treatment initiation according to current guidelines
- The assessment of the impact of non-invasive arterial stiffness estimation on target organ damage and patient's CV prognosis
- The definition of the normalcy thresholds for PWV, AI and other current and future indices derived from PWA, in hypertensive subjects, according to outcome data
- The definition of the relationship between arterial stiffness, blood pressure absolute level and blood pressure variability, and outcomes
- The setup of a worldwide network of centers performing ambulatory PWA, and the validation and promotion of the use of such technique for hypertension screening and follow-up
- The provision of evidence on the clinical relevance of non-invasive arterial stiffness assessment, in order to favor the inclusion of such evaluation in recommendations on hypertension management

8.0 OVERALL PROJECT DESIGN AND PLAN

International, multicenter, observational, prospective project

9.0 SELECTION OF PROJECT POPULATION

The registry will include data from subjects fulfilling the inclusion criteria and whose data are contained in

existing databases collected by the participating centers and who are regularly followed-up at the center. New subjects can be enrolled for this project, but they must be submitted to ABPM because it is required for evaluating their hypertension status, according to current recommendations.
While the present document contains the initial list of participating countries (Italy and Russia) and centers, other centers willing to participate in the project and able to provide a significant contribution may be accepted also after the project has initiated.

9.1 Inclusion criteria

- Male and female subjects
- Age ≥18 years
- Subjects referred to routine diagnostic evaluation for hypertension or established hypertensive subjects
- ABPM performed for clinical reasons with a BPLab device
- The minimum validity requirements for inclusion of the ABPM in the study are:
  - Interval between measurements not exceeding 30 minutes
  - At least 70% of expected number of readings
  - At least 20 valid readings during the day-time and 7 during the nigh-time
- Availability of individual measurements for ABPM on a .bpw file (BPLab format) or data directly downloaded on the telemedicine platform of the study (see below)
- Availability of basic clinical information (as detailed in Section 14.1):
- Availability of a signed informed consent form

9.2 Exclusion criteria

- Age <18 years
- Atrial fibrillation, frequent ectopic beats, second or third degree atrioventricular blocks, or other conditions which might make difficult or unreliable the automatic blood pressure measurement with the oscillometric technique
- Upper arm circumference <22 cm
- Pregnancy

10.0 PROJECT ACTIVITIES

10.1 Creation of research network and data collection

Data contained in existing electronic databases or data of newly enrolled subjects fulfilling the inclusion criteria will be uploaded/entered on the study website. These data will include ABPM measures (blood pressure and arterial stiffness) obtained with a BPLab device and clinical data. ABPM data will be provided as .bpw files or directly uploaded by linking the ABPM device to the website through a personal computer. 24-hour ambulatory blood pressure recordings will be done in accordance with the procedures described in the *Appendix 1*.
Data collection will be ensured by a dedicated web-based telemedicine platform, including an electronic case report form (e-CRF). ABPM data will be transmitted to the project website and analyzed in real-time with production of an electronic report sent by e-mail to the Investigator and available on the website. Only ambulatory blood pressure data collected with a BPLab monitor will be included in the system. The e-CRF will allow collection of patient's clinical data, such as family history, anthropometric data, habits, past and current diseases, therapies, office BP, and laboratory tests, including evaluation of target organ damage.
More than one ABPM recording may be performed in the same patient if deemed necessary by the physician in charge. They will be uploaded in the telemedicine system and complemented by an update of clinical information, as mentioned above. The project does not involve any type of intervention related to the study and the physicians will manage the patients included in the registry according to the requirements of clinical practice and current guidelines. However, as recommended by current guidelines, each patient will be followed-up with visits occurring at regular intervals (ideally every 6 months, and not less than once a year). The physicians will also be free to use the ambulatory blood pressure data in the clinical management of the patients.

The telemedicine system will allow a) standardized and centralized data collection, b) data validation by experts and counselling to remote centers, c) setup and maintenance of the Registry.

10.2 ABPM data analysis

Principal derived ABPM variables and arterial stiffness measures will be calculated immediately once the data are input in the database and their adequate quality is verified. Mean blood pressure and heart rate values, PWV and AI will be computed for 24-hour, day-time and night-time by averaging the all the individual readings for the subperiod in question. Measures of blood pressure variabilities (weighted standard deviation and average real variability) will also be computed based on individual readings.
Other variables of interest (e.g. nocturnal blood pressure fall, morning surge, etc.) will be subsequently defined in the framework of sub-analyses based on the registry data and calculated according to the procedures specifically defined.

10.3 Dissemination activities

An important part of activities in this project will be aimed at disseminating the knowledge on correct use of ambulatory blood pressure and arterial stiffness estimation in clinical practice and in research and thus at achieving a possibly standardized and widespread use of this integrated technology in the participating centers. In order to achieve these aims the project, apart from data collection, will involve the following activities:

- Exchange of knowledge between participating centers already expert in ABPM use and in arterial stiffness measurement. This will be mainly achieved by cooperation of investigators in preparing a possibly unified methodology of ABPM data collection and analysis and by jointly addressing methodological issues that may arise during the project.
- Performance of studies aimed at optimizing a possible clinical application of non-invasive ambulatory arterial stiffness estimation, based on data collected in the Registry
- Providing instructions on appropriate ambulatory arterial stiffness monitoring methodology to other physicians. Indeed, an important feature of the project will be to actively involve intermediate level centers, not necessarily expert in ABPM use and arterial stiffness determination. A major task of the consortium will be to provide these participants with an accurate information on correct methodology and interpretation of such data, in order to support them in case of difficulties and to monitor the correctness of the use of the methodology in these centers during the project
- Preparation of specific recommendations on the use and clinical application of ABPM integrated with arterial stiffness evaluation
- Cooperation with international and national scientific societies in the area related to ABPM and arterial stiffness monitoring. The majority of the VASOTENS Scientific Committee members will be selected among active members of international and national hypertension societies, including the membership in the bodies specifically dedicated to blood pressure and arterial stiffness measurement. This will facilitate the dissemination of information on the project and its findings and will also allow an interaction with writing committees involved in the preparation of guidelines pertinent to this area

11.0 STATISTICAL METHODS

Basic descriptive statistics of the entire registry with calculation of absolute and relative frequencies for categorical variables and calculation of average value, standard deviation, minimum and maximum for continuous variables will be calculated.
Standard variables (systolic and diastolic blood pressure, pulse pressure and heart rate), central systolic and diastolic blood pressure, and arterial stiffness indices (PWV and AI) will be averaged for the whole 24-hour period, and separately for the day-time and night-time subperiods. Additionally, hourly averages will be computed, and in case of blood pressure, variability will be evaluated by calculation of weighted standard deviation and average real variability.
Relationship between blood pressure and arterial stiffness estimates and organ damage and prognosis will be evaluated.

Other variables of interest will be defined in the course of the study and calculated subsequently according to the procedures defined in a specific statistical analysis plan.
The occurrence of any cardiovascular event (death or hospitalization for congestive heart failure, myocardial infarction, angina, stroke or cerebrovascular accident, renal failure, etc.) during the study will be evaluated by the Kaplan-Meier method. Time-to-event curves will be drawn and the survival analysis will be performed according to the Cox proportional hazard model, which will allow to analyze predictors of outcomes.
Analysis will be performed periodically: the first will be done at the end of the first year of follow-up of all the recruited subjects.

12.0 INVESTIGATORS AND PROJECT ADMINISTRATIVE STRUCTURE

12.1 Project Coordinators

The Promoter of the project and Scientific Coordinator is:

Dr. Stefano Omboni
Clinical Research Unit
Italian Institute of Telemedicine
Via Colombera 29
21048 Solbiate Arno (Varese)
Italy
Tel +39 0331 984529
Fax +39 0331 984530
stefano.omboni@iitelemed.org

Study co-coordinator is:

Dr. Igor Posokhov
Hemodynamic Laboratory
PO Box 69
603009 Nizhniy Novgorod
Russia
Tel +7 831 2961414
Fax +7 831 2961415
igor@posohov.ru

12.2 Scientific Committee and other project structures

The Project Coordinators will be supported by the following structures.

12.2.1 Steering Committee

The Steering Committee will directly coordinate the project activities and prepare the preliminary versions of the project documents to be subsequently presented to the Scientific Committee. The Steering Committee will be formed by the Project Coordinators and by the individual coordinator of each country (National Coordinator). Members of the Steering Committee are:

Stefano Omboni (Italy), Gianfranco Parati (Italy), Igor Posokhov (Russia), Anatoli Rogoza (Russia).

12.2.2 Scientific Committee

The main tasks of the Scientific Committee will be the design of the project, the selection of participating doctors, check and approval of the protocol, of its appendices and possible amendments, supervision of and support to Investigators, evaluation of the results and preparation of manuscripts based on project results. The Scientific Committee will be formed by the Project Coordinators, the National Coordinators and by no

more than one major local investigator.

Stefano Omboni (Italy), Gianfranco Parati (Italy), Igor Posokhov (Russia), Anatoli Rogoza (Russia), Yulia Kotovskaya (Russia)

12.2.3 National Coordinators

The National Coordinators will coordinate local investigators and stimulate recruitment and data collection in each country. They will also organize, when feasible, local investigators' meeting.

12.2.4 Scientific and Organizing Secretariat

The Contract Research Organization (CRO) of the study will be Docleader Ltd. located in Solbiate Arno (Varese), Italy. When requested, this company will assist the Promoter and Coordinators in all the scientific and practical aspects related to the study, e.g. protocol and appendices definition, organization of investigators' meeting, contacts with the investigators, data management and analysis, reporting, etc. (see Section 12.4)

12.3 Investigators

This multicenter international project will involve investigators from Hypertension Centers worldwide. Initially two countries will be involved in the studies (Italy and Russia).
Basic technical requirements for the inclusion of a center in the project will be:

- The availability in the facility of a BPLab ABPM monitor
- The ability to perform proper 24-hour ABPM
- The potential for providing and properly following-up the number of patients required by the protocol
- The availability of Internet connection, regular access to the web and human resources to upload ABPM and clinical data

The investigator will need to have a medium-high level of expertise in informatics.
Given the observational nature of the project, it will not foresee any costs for the doctors and health insurance system and will not cause implementation of additional diagnostic examinations or changes in the use of any specific class of antihypertensive drugs.

12.4 CRO

Upon request, the CRO of the project will assist the Promoter in some of the activities related to the study (e.g. protocol finalization and formatting, training of the investigators in the project procedures, data management and analysis, preparation of statistical reports, etc.)
The CRO will have the responsibility to take all the necessary measures to ensure proper conduct of the project regarding the ethical aspects, adherence to the protocol, and the integrity and validity of data recorded in the e-CRF.

13.0 ETHICS

13.1 Independent Ethics Committee or Institutional Review Board

The data collection can be started in each center only after approval or notification (depending on local laws) of the project protocol Independent Ethics Committees.

13.2 Subject information and consent

The investigator must obtain written informed consent from each patient (*Appendix 2*), before submitting him or her to any procedure of the project.
Each patient must provide written informed consent after receiving information and explanations about the

objectives, commitments, rights and responsibilities faced by his/her participation in the project. In particular, the patient must be informed:

- That this is a research project
- About the objectives of the project
- About the project procedures
- About the free and voluntary participation in the project, and the possibility to get more information on the project from the investigator in charge of the project
- That anonymity of the patient will be kept on the official documentation of the project
- About the methods for the processing of personal data

The information will be provided to the patient through an information sheet (*Appendix 2*) and verbally by the investigator.
The written informed consent of the patient should be documented on the appropriate form, which must be dated and signed by the subject. According to local laws, the patient might also be asked to sign a separate form for the processing of personal data. The patient who will not provide the written authorization will not participate in the project. In case of subjects legally incapable or with restriction, consent must be provided by the legal representative.
The original signed informed consent obtained from the patient will be retained by the investigator in the patient's file at the project center, while one copy will be delivered to the patient.

14.0 DATA MANAGEMENT

14.1 Electronic Case Report Form (e-CRF)

Data will be collected for each patient by an e-CRF located on a website. The e-CRF will allow the collection of the main demographic and clinical data including the patient's medical history and concomitant therapies. All data must accurately reflect the patients' view, or be an expression of their knowledge about their health.
The following data will be entered fin the e-CRF, for each subject and visit:

- Age
- Gender
- Height
- Weight
- Ethnicity
- Superficial distance between jugulum and symphysis (surrogate of aortic length)
- Waist circumference
- Smoking status
- Alcohol drinking
- Coffee or tea drinking
- Dyslipidemia (yes/no and indication on treatment)
- Diabetes (yes/no and indication on treatment)
- Diagnosis of hypertension (yes/no and indication on treatment)
- Family history of premature CV disease
- Medical history, with particular regard to previous and/or concurrent CV diseases
- Office blood pressure and heart rate obtained in the same treatment condition as ABPM
- Left ventricular mass index (LVMI) at echocardiogram
- When available, diameter of the aorta (aortic annulus, root and sinotubular junction) and/or cardiac output, assessed by the echocardiogram
- Intima-media thickness (IMT) at carotid ultrasonography
- ECG (indication on left ventricular hypertrophy, Sokolow–Lyon and Cornell index)
- When available, ankle-brachial index

- Microalbuminuria and serum creatinine (calculation of estimated glomerular filtration rate - eGFR)
- When available, pulse wave velocity (PWV) augmentation index (AI) and central blood pressure taken during the office visit with a validated device different for BPLab Vasotens (e.g. Sphygmocor or Complior)

Access to the e-CRF will be granted through authentication with ID and password. The investigator will be responsible for the faithful transcription of the results of any laboratory or instrumental diagnostic tests. If changes have to be made to data, they will be overwritten with the correct ones. The system will keep record of the date and time of the corrections.
The data entered in the e-CRF will be stored in a database residing on a webserver. When entering data in the e-CRF the system will automatically check their congruence. A further data check will be made on the database in order to highlight missing data, or compilation errors, inconsistencies, protocol violations. The list of these errors will be discussed with the investigator, and appropriate changes agreed with him/her.
The blood pressure and arterial stiffness measurements made with the BPLab blood pressure monitor will be uploaded electronically by the telemedicine system onto the e-CRF.

14.2 Project monitoring

Given its observational nature, no formal monitoring is foreseen for this project. However, electronic data verification could be done by the monitor and data manager, who will get in touch with the investigators, via e-mail, fax or phone, and when needed may ask the investigator to correct the erroneous data or complete missing data on the e-CRF.
Each investigator will be required to keep records of the project in a file located at the project center. The investigator is asked to keep a paper or computer file of the patients enrolled in the project, but also of those assessed but not included in the project.
The Promoter might eventually ask the CRO to visit the center and will thus have free access to the documentation of the project in order to:

- Check the progress of the project
- Verify adherence to the protocol procedures
- Discuss any problems
- Review the adequacy of the completion of the e-CRF and that its content complies with the source data (in this case the patient's personal data will still be kept confidential)

The verification of source documentation may also be made in the course of an audit by the Promoter in order to ensure the validity of the data, or during the inspection by the competent authorities, depending on local rules. Personal data of the patient will still be kept confidential.

14.3 Data quality control and assurance

The investigator agrees to participate in the project in full adherence to the present protocol and to verify and check that the information provided on the e-CRF is as precise and accurate as possible.
The procedures for data monitoring and verification will be ensured by the presence of logical checks and range (defined a priori) for the different variables and by automatic identification of inconsistencies by the software used to manage the database. The controls and related corrections can be made on the e-CRF directly by the investigator on the website.

15.0 INSURANCE

Because of its observational nature, this project does not require insurance, apart from that already required for normal clinical practice and available at each general practice’s or specialist’ office.

16.0 DISCONTINUATION OF THE PROJECT

The Promoter reserves the right to discontinue the project at any time. This decision will be communicated in writing to the investigator. Similarly, if the investigator decides to withdraw from the project he/she must give immediate written notice to the Promoter.

17.0 STATEMENT OF CONFIDENTIALITY

All documentation related to the project will be provided to the investigator and his coworkers by the Promoter under conditions of confidentiality. None of these documents may be disclosed to third parties not directly involved in the project without the written permission of the Promoter.
The investigator must ensure the anonymity of individual patients.
All materials, information (verbal or written) and unpublished documents provided to the investigators, including this protocol are the exclusive property of the Promoter. This material or information (either global or partial) cannot be delivered or disclosed by the investigator or any other person of his or her group to any unauthorized person without a formal written consent of the Promoter.
The investigator will consider confidential all information received, acquired or derived during the project and take all necessary measures to ensure their confidentiality in accordance with the privacy requirements and local privacy laws.

18.0 REPORTING

Detailed reports of the study results will be published, starting form the end of the first year of follow-up. The investigator agrees that the results of this study can be verified by national and/or international quality control authorities. To do so, if requested, the investigator must provide to these authorities all the information requested.

19.0 DATA ACCESS AND PUBLICATION POLICY

All unpublished material (for example the protocol and the e-CRF) provided to the investigator is confidential and cannot be disclosed in any way without the written approval of the Scientific Coordinators.
All data and results and all intellectual property rights derived from the project are the property of the Promoter.
Any publication or oral presentation concerning the project, even when regarding partial results, must be approved and authorized by the Promoter.
At each point of the project each member of the Scientific Committee can propose the performance of an analysis based on the data contained in the registry. The draft of the analysis plan will be discussed among the Scientific Committee members and undergo the final approval by the Scientific Coordinators and the Steering Committee. The author of the proposal will coordinate the data analysis, which will be performed centrally.
The authors' list for each publication will include the proponent(s) (as the first authors), all members of the Steering Committee, and up to three people most directly involved in the performance of the study. The authors list will include the statement "on behalf of VASOTENS Investigators" and the complete list of Scientific Committee members and Investigators will be provided at the end of the paper. This policy may be modified in individual cases upon agreement of all interested sides.

20.0 PROTOCOL AMENDMENTS

This protocol and appendices form an integral part of this document.
After the protocol is negotiated and signed by the parties no modification can be made by the Promoter, nor by the Coordinators or by the investigators, without agreement among the parties. Any agreed changes will be ratified in writing by the Promoter and Coordinator and annexed to this protocol.
Any amendment will be reported to the Ethics Committee.

21.0 REFERENCE LIST

1 Laurent S, Cockcroft J, Van Bortel L, Boutouyrie P, Giannattasio C, Hayoz D, Pannier B, Vlachopoulos C, Wilkinson I, Struijker-Boudier H; European Network for Non-invasive Investigation of Large Arteries. Expert consensus document on arterial stiffness: methodological issues and clinical applications. Eur Heart J 2006; 27: 2588-2605.

2 Vlachopoulos C, Aznaouridis K, Stefanadis C. Prediction of cardiovascular events and all-cause mortality with arterial stiffness: a systematic review and meta-analysis. J Am College Cardiol 2010; 55: 1318-1327.

3 McEniery CM, Cockcroft JR, Roman MJ, Franklin SS, Wilkinson IB. Central blood pressure: current evidence and clinical importance. Eur Heart J 2014; 35: 1719-1725.

4 ESH/ESC Task Force for the Management of Arterial Hypertension. 2013 Practice guidelines for the management of arterial hypertension of the European Society of Hypertension (ESH) and the European Society of Cardiology (ESC): ESH/ESC Task Force for the Management of Arterial Hypertension. J Hypertens 2013; 31: 1925-1938.

5 O'Rourke MF, Adji A. Noninvasive studies of central aortic pressure. Curr Hypertens Rep 2012;14:8-20.

6 Baulmann J, Schillings U, Rickert S, Uen S, Düsing R, Illyes M, Cziraki A,Nickering G, Mengden T. A new oscillometric method for assessment of arterial stiffness: comparison with tonometric and piezo-electronic methods. J Hypertens 2008; 26: 523-528.

7 Trachet B, Reymond P, Kips J, Swillens A, De Buyzere M, Suys B, Stergiopulos N, Segers P. Numerical validation of a new method to assess aortic pulse wave velocity from a single recording of a brachial artery waveform with an occluding cuff. Ann Biomed Eng 2010; 38: 876-888.

8 Hametner B, Wassertheurer S, Kropf J, Mayer C, Eber B, Weber T. Oscillometric estimation of aortic pulse wave velocity: comparison with intra-aortic catheter measurements. Blood Press Monit 2013; 18: 173-176.

9 Posokhov IN. Pulse wave velocity 24-hour monitoring with one-site measurements by oscillometry. Med Devices (Auckl) 2013; 6: 11-15.

10 Ageenkova OA, Purygina MA. Central aortic blood pressure, augmentation index, and reflected wave transit time: reproducibility and repeatability of data obtained by oscillometry. Vasc Health Risk Manag 2011; 7: 649-656.

11 Luzardo L, Lujambio I, Sottolano M, da Rosa A, Thijs L, Noboa O, Staessen JA, Boggia J. 24-h ambulatory recording of aortic pulse wave velocity and central systolic augmentation: a feasibility study. Hypertens Res 2012; 35: 980-987.

12 Omboni S, Posokhov IN, Rogoza AN. Evaluation of 24-Hour Arterial Stiffness Indices and Central Hemodynamics in Healthy Normotensive Subjects versus Treated or Untreated Hypertensive Patients: A Feasibility StudyVolume. Int J Hypertens 2015:601812.

13 Zhang Y, Kollias G, Argyris AA, Papaioannou TG, Tountas C, Konstantonis GD, Achimastos A, Blacher J, Safar ME, Sfikakis PP, Protogerou AD. Association of left ventricular diastolic dysfunction with 24-h aortic ambulatory blood pressure: the SAFAR study. J Hum Hypertens 2014 Nov 13 [Epub ahead of print].

14 Protogerou AD, Argyris AA, Papaioannou TG, Kollias GE, Konstantonis GD, Nasothimiou E, Achimastos A, Blacher J, Safar ME, Sfikakis PP. Left-ventricular hypertrophy is associated better with

24-h aortic pressure than 24-h brachial pressure in hypertensive patients: the SAFAR study. J Hypertens 2014;32:1805-1814.

22.0 APPENDICES

Appendix 1 – Ambulatory blood pressure monitoring procedures

1. ABPM Device

Twenty-four hour ambulatory blood pressure monitoring (ABPM) will be performed with the BPLab device, which has been successfully validated for both blood pressure and arterial stiffness measurement.

2. ABPM settings

Before performing the monitoring the device should be set up according to the following indications:

- Interval between measurements must not exceed 30 minutes during the whole 24-hours (2 readings per hour): ideally the device should be set to measure blood pressure every 20 minutes during the day-time (3 readings per hour) and 30 minutes during night-time (2 readings per hour).
- Daytime/activity period should be defined as 7 a.m. – 11 p.m. or according to the diary
- Night-time/sleep period should be defined as 11 p.m. – 7 a.m. or according to the diary
- Values display should be turned off
- Pre-measurement acoustic signal should be turned off at night, in order not to disturb patient's sleep
- Criteria for identifying valid readings should be set as follows:

  - Pulse pressure between 10 and 150 mm Hg
  - Systolic blood pressure between 50 and 300 mm Hg
  - Diastolic blood pressure between 40 and 150 mm Hg
  - Systolic blood pressure always greater than diastolic blood pressure
  - Heart rate between 40 and 150 bpm

3. Monitoring conditions

Recording should start preferably between 8 a.m. and 11 a.m. ABPM cuff should be placed on the nondominant arm, the lower edge 2 cm above elbow bend; the bladder position as indicated by the manufacturer. One or two test readings should be triggered manually before the device is activated for automatic measurements. Conventional blood pressure and heart rate should be measured and recorded at the time of ABPM placement. Patients should be instructed to keep the arm still and to avoid any movement during each automatic blood pressure measurement. Patients will be free to attend their usual daily activities during ABPM (avoiding strenuous exercise). Patients should complete a diary in which daily activities such as time of sleeping, time of meals, etc. will be reported together with the time of occurrence of unusual events or poor night sleep quality. The patient should come to the outpatient clinic on the second day of the recording (after at least 24 hours) to remove the monitor.

4. ABPM data handling and quality verification

Shortly after the device removal the recording should be downloaded to PC using the telemedicine web platform of the project.
The Investigator should verify each recording for compliance with quality criteria. The quality of a recording will be considered acceptable if:

- Interval between measurements not exceeding 30 minutes during the whole 24-hours (2 readings per hour)
- Recording duration of at least 24 hours
- At least 70% of expected number of readings
- At least 20 valid readings during the day-time and 7 during the nigh-time

If a blood pressure recording does not fulfill the above mentioned criteria it should be repeated.
The Investigator should make sure to receive the filled-in patient diary form and should input the required information in the online ABPM diary form.

Appendix 2 – Patient information sheet and informed consent form

INTERNATIONAL REGISTRY FOR AMBULATORY BLOOD PRESSURE AND ARTERIAL STIFFNESS TELEMONITORING (VASOTENS REGISTRY)

Patient number:	Initials:	Date: / /
		DAY MONTH YEAR

PATIENT INFORMATION SHEET
version 1.0 28/01/2015

The present study and related procedures are carried out for purposes of clinical research and the following information is intended to illustrate the study purpose. Your participation in this study will be voluntary.

PURPOSE OF THE RESEARCH

The aim of this study is to determine whether your blood pressure is well controlled or not, by using an automatic method of blood pressure measurement over the 24-hours.
The study will include approximately 3,000 patients with high blood pressure or hypertension, controlled (i.e. a pressure below 140/90 mmHg) or uncontrolled (i.e., a pressure equal to or greater than 140/90 mmHg), treated or untreated. All patients will be submitted to a 24-hour ambulatory blood pressure monitoring with an automated device, provided free by the doctor and to be returned to the doctor after 24-hours of recording. This instrument will record your blood pressure, and estimate arterial stiffness, at intervals of 20-30 minutes throughout the 24-hours. At the end of the recording your doctor will examine the blood pressure values and, if necessary, will modify treatment accordingly.
Recent studies suggest that when blood pressure is measured in such a way, clinicians may better define the degree of blood pressure control and improve hypertension treatment. With this study we try to confirm this hypothesis on a large scale, worldwide. In addition, the estimation of arterial stiffness through the analysis of pulse wave measured with the device will allow to add new potentially relevant information of the arterial function, which is often impaired in hypertension. If, as we think, the results of the study confirm our hypothesis, we will have further data supporting the use of ambulatory blood pressure monitoring as an important and useful tool to better define the blood pressure and arterial function of hypertensive patients and to make a more accurate diagnosis of this condition.
The study does not foresee the use of drug treatment, which may, however, be modified or started by your doctor as a consequence of the result of the monitoring. If such changes are deemed necessary, they will not be done within the frame of the study and will be made according to the usual clinical practice.

PROCEDURES OF THE RESEARCH

The study foresees one or more visits during which the doctor will measure your blood pressure and then apply an upper arm cuff connected to a blood pressure measuring device which will measure your blood pressure automatically. The device will be fixed at your waist by a belt. You have to carry about this device for 24-hours, taking care not to damage it and you will come back to the doctor at the end of this period to unfit the device. During each automatic measurement, which will take place every 20-30 minutes, you must keep the arm extended along your body and stand still and in silence to allow the device to reliably and accurately measure your blood pressure.
During the fitting of the device and before starting the recording the doctor will also question you about your health, especially in terms of cardiological problems or disease, and any medications you have taken. You will also be asked to provide recent laboratory, blood and urine tests, and results of other laboratory tests, which may be performed during the visit or in the preceding or following days.

BENEFITS

By participating in this study, you will help to better define the importance of using the technique of 24-hour ambulatory blood pressure monitoring in the determination of actual blood pressure control and arterial function of hypertensive patients. This might eventually help in defining the optimal treatment of this condition. You may not have immediate benefits or have no direct benefit from the procedure you are subjected to.

POSSIBLE RISKS

There are no risks associated with participating in this study. During the study period the physician responsible for carrying out this study will constantly monitor your health status and intervene quickly in case of occurrence of any adverse event.

REIMBURSEMENTS

The participation in the study is completely free. You shall not bear any cost for the procedures of the study, which are considered routine in patients with hypertension. No compensation is provided for participation in this study.
Your participation in the study as a patient is covered by an insurance provided for routine clinical practice.

VOLUNTARY PARTICIPATION AND RIGHT TO REFUSE OR WITHDRAW

You agree to voluntarily participate in this study and will therefore have the right to withdraw at any time, without jeopardizing medical care or treatment to which you are entitled.
In your interest, the doctor may exclude you from the study if you do not comply with the study procedures, or whether in your interest, he considers to withdraw you from the study. If you decide to participate in this study, you will have to visit the doctor to apply and remove the automatic blood pressure monitor. In case additional information relevant to your participation in this study becomes available, you will be promptly advised by the doctor.

CONFIDENTIALITY

With this research, something out of the ordinary is being done in your community. It is possible that if others in the community are aware that you are participating, they may ask you questions. We will not be sharing the identity of those participating in the research.
The information that we collect from this research project will be kept confidential. Information about you that will be collected during the research will be put away and no-one but the researchers will be able to see it. Any information about you will have a number on it instead of your name. Only the researchers will know what your number is and we will lock that information up with a lock and key. It will not be shared with or given to anyone except the study Promoter or persons designated by him to follow the study.

WHO TO CONTACT

You may take your time to study this information document and ask any questions on points that seem unclear.
At any time, you may refer to the doctor responsible for this study for any question regarding the study,
Dr. ____________________________________ telephone number ______________________

INTERNATIONAL REGISTRY FOR AMBULATORY BLOOD PRESSURE AND ARTERIAL STIFFNESS TELEMONITORING (VASOTENS REGISTRY)

Patient number: Initials: Date: / /

DAY MONTH YEAR

INFORMED CONSENT FORM

version 1.0 28/01/2015

Declaration of consent

I have read the foregoing information, or it has been read to me. I declare that I have understood all the information relating to the present clinical research, and that I have had the opportunity to ask questions about it and any questions that I have asked have been answered to my satisfaction.
I consent voluntarily to participate in the study, knowing that such participation is voluntary and that I can withdraw at any time.
I agree to provide laboratory examinations and blood tests performed before entering the study.
By signing this form I agree also to handle my personal data for research purposes to the extent and in the manner indicated in the information sheet provided to me with the present document.

I will receive a copy of the present informed consent form, after signing.

PARTICIPANT'S SURNAME AND NAME ____________________
(in block letters)

SIGNATURE ____________________ DATE ____________
(day/month/year)

I have accurately read out the information sheet to the potential participant, and to the best of my ability made sure that the participant understood the procedures of the study.
I confirm that the participant was given an opportunity to ask questions about the study, and all the questions asked by the participant have been answered correctly and to the best of my ability. I confirm that the individual has not been coerced into giving consent, and the consent has been given freely and voluntarily.
A copy of this Informed Consent Form has been provided to the participant.

INVESTIGATOR'S SURNAME AND NAME ____________________
(in block letters)

INVESTIGATOR'S SIGNATURE ____________________ DATE ____________
(day/month/year)

Appendix 3 – Investigator's approval page

INVESTIGATOR'S APPROVAL PAGE

I confirm that I have examined the protocol of the project "INTERNATIONAL REGISTRY FOR AMBULATORY BLOOD PRESSURE AND ARTERIAL STIFFNESS TELEMONITORING (VASOTENS REGISTRY)" and that I have discussed the project objectives and contents with the Promoter or the CRO.

I understand that all information concerning the project provided to me by the Promoter is confidential. This information includes the protocol, the e-CRF and all other possible documents.

I understand that any change in this protocol must be approved in writing by the Promoter, the Study Coordinator and the Ethics Committee before implementation, except where necessary to eliminate apparent immediate hazard to the patients.

I confirm that I will conduct the project according to this protocol and laws and regulations in the Country where the research is to be conducted.

I confirm that I am informed of the need to record retention and that no data can be destroyed without the written consent of the Promoter.

I agree to conduct the project according to this protocol and to comply with its demands.

A possible termination or suspension of the project decided by the Promoter will be notified to me in writing.

Similarly, in case I decide to withdraw from the project, I will give prompt written notice to the CRO, representing the Promoter.

Principal Investigator

Name and surname ____

Institution ____

Address ____

Date ______________________________ Signature __________________________________

The CRO on behalf of the Promoter

Name and surname ____

Date ______________________________ Signature __________________________________
