# Supplementary material for: Vascular Health Assessment of The Hypertensive Patients (VASOTENS) Registry: Study Protocol of an International, Web-Based Telemonitoring Registry for Ambulatory Blood Pressure and Arterial Stiffness
Source: JMIR Res Protoc. 2016 Jun 29;5(2):e137. doi: 10.2196/resprot.5619 (PMC4945820; doi:10.2196/resprot.5619)
Supplement: Multimedia Appendix 3 [file resprot_v5i2e137_app3.pdf]

THE VASOTENS REGISTRY STUDY GROUP

Study coordinator
Stefano Omboni (Italy)

Study Co-coordinator
Igor Posokhov (Russia)

Steering Commitee
Stefano Omboni (Italy), Gianfranco Parati (Italy), Igor Posokhov (Russia), Anatoli Rogoza (Russia)

Scientific Committee
Stefano Omboni (Italy), Gianfranco Parati (Italy), Igor Posokhov (Russia), Anatoli Rogoza (Russia), Yulia Kotovskaya (Russia)

Investigators
Argentina: Gabriel Waisman, Pedro Forcada.
Armenia: Parounak Zelveian.
Australia: Alberto Avolio, Mark Butlin, Edward Barin
Italy: Stefano Omboni, Gianfranco Parati, Giuseppe Mulè, Lorenzo Ghiadoni, Rosa Maria Bruno, Riccardo Sarzani, Emma Espinosa, Massimo Volpe, Carmine Savoia, Giuliano Tocci, Claudio Borghi, Giuseppe Schillaci, Giacomo Pucci, Maria Lorenza Muiesan, Anna Paini, Damiano Rizzoni.
Mexico: Ernesto Cardona Muñoz , Carlos Ramos, Adrian Alanis.
Portugal: Telmo Pereira, João Manuel Peixoto Maldonado
Romania: Ioan Tilea, Andreea Varga
Russia: Igor Posokhov, Anatoli Rogoza, Dmitriy Volkov, Yulia Kotovskaya, Zhanna Kobalava, Iana Orlova, Natalya Kurlykina, Alexandra Konradi, Oxana Rotar, Alexander Orlov, Elena Fedorova, Viktoria Korneva, Tatyana Kuznetsova, Natalya Kulikova, Elena Grigoricheva, Vitaly Evdokimov, Anastasiya Kuznetsova, Elena Zheleznyak, Vitaly Barkan, Irina Minyukhina.

Data Management

Stefano Omboni (Italy), Igor Posokhov (Russia)
